# Kinetics of Methylation by EcoP1I DNA Methyltransferase

**DOI:** 10.4061/2010/302731

**Published:** 2010-07-15

**Authors:** Shivakumara Bheemanaik, Srivani Sistla, Vinita Krishnamurthy, Sampath Arathi, Narasimha Rao Desirazu

**Affiliations:** Department of Biochemistry, Indian Institute of Science, Bangalore 560 012, India

## Abstract

EcoP1I DNA MTase (M.EcoP1I), an N^6^-adenine MTase from bacteriophage P1, is a part of the EcoP1I restriction-modification (R-M) system which belongs to the Type III R-M system. It recognizes the sequence 5′-AGACC-3′ and methylates the internal adenine. M.EcoP1I requires Mg^2+^ for the transfer of methyl groups to DNA. M.EcoP1I is shown to exist as dimer in solution, and even at high salt concentrations (0.5 M) the dimeric M.EcoP1I does not dissociate into monomers suggesting a strong interaction between the monomer subunits. Preincubation and isotope partitioning studies with M.EcoP1I indicate a kinetic mechanism where the duplex DNA binds first followed by AdoMet. Interestingly, M.EcoP1I methylates DNA substrates in the presence of Mn^2+^ and Ca^2+^ other than Mg^2+^ with varying affinities. Amino acid analysis and methylation assays in the presence of metal ions suggest that M.EcoP1I has indeed two metal ion-binding sites [^358^ID(x)_n_ … ExK^401^ and ^600^DxDxD^604^ motif]. EcoP1I DNA MTase catalyzes the transfer of methyl groups using a distributive mode of methylation on DNA containing more than one recognition site. A chemical modification of EcoP1I DNA MTase using *N*-ethylmaleimide resulted in an irreversible inactivation of enzyme activity suggesting the possible role of cysteine residues in catalysis.

## 1. Introduction

DNA methyltransferases (MTases) catalyze the transfer of methyl groups to DNA from *S*-adenosyl-L-methionine (AdoMet) to form methylated DNA and *S*-adenosyl-L-homocysteine (AdoHcy). In prokaryotes, DNA methylation participates in DNA strand discrimination during postreplicative mismatch repair, protection against phages, gene regulation, cell cycle control, bacterial virulence, transcription, and control of DNA replication [1]. The distinction of self and nonself DNA is associated with restriction-modification (R-M) systems, which function as host defense mechanisms against the infection of bacteria by bacteriophages [1]. DNA MTases are known to modify either adenine at the N^6^ position or cytosine residues at the N^4^ or C^5^ positions [2]. Based on the position of methyl groups transferred to DNA, DNA MTases are classified into two classes: exocyclic amino MTases and endocyclic MTases. Exocyclic DNA MTases methylate the amino group of cytosine or adenine to form N^4^-methylcytosine or N^6^-methyladenine while endocyclic MTases methylate cytosine residues at the C^5^ position. A structure-guided sequence analysis of exocyclic and endocyclic DNA MTases showed that nine conserved motifs (motifs I-VIII and X) are present suggesting that DNA MTases are closely related to one another [3].

EcoP1I belongs to the Type III R-M system encoded by the prophage P1 that infects *Escherichia coli* [1]. It contains two subunits: Mod and Res. Although the EcoP1I restriction enzyme is an efficient MTase, a separate MTase (product of the *mod* gene) can be purified with methylation as the only enzymatic activity. EcoP1I DNA methyltransferase (M.EcoP1I) catalyses the transfer of a methyl group from AdoMet to the second adenine in the recognition sequence 5′-AGACC-3′ to form N^6^-methyladenine in the presence of metal ions [4]. One of the interesting features of Type III R-M systems is that they recognize asymmetric recognition sequences and methylate only one strand [5]. Interestingly, M.EcoP1I binds to both double- and single-strand DNA substrates containing the asymmetric recognition sequence 5′-AGACC-3′ and methylates them with almost similar efficiency [6]. Based on the linear arrangement of conserved motifs, M.EcoP1I belongs to the *β*-subgroup of exocyclic amino MTases (catalytic motif-target recognition domain-AdoMet binding motif). Mutational analysis of the AdoMet-binding motif and the catalytic motif of M.EcoP1I abolished methylation activity suggesting the importance of the motifs in AdoMet binding and transfer of the methyl group to DNA [7]. Unlike M.EcoP1I, EcoP15I DNA MTase (M.EcoP15I), the other well-characterized-MTase from the Type III R-M system, methylates double-strand DNA in the presence of Mg^2+^ and does not methylate single-strand DNA [8]. Mutations in the metal-binding motif, amino acid residues 355–377 in M.EcoP15I, resulted in a loss of methylation activity. It was shown that the Mg^2+^ ion was required for base flipping during the methyl transfer reaction [8]. This study reports the order of substrate binding, two metal ion requirement, and mode of methylation by EcoP1I DNA MTase.

## 2. Materials and Methods

### 2.1. Bacterial Strains and Plasmids

*Escherichia coli* DH5*α* (F^−^ 80d*lac*Z M15 (*lac*ZYA-*arg*F) U169 *rec*A1 *end*A1*hsd*R17(r_k_^−^, m_k_^+^) *pho*A*sup*E44 ^−^ *t* *h* *i*-1 *gyr*A96 *rel*A1) cells were used for the isolation of DNA. Proteins were expressed in *E. coli* BL21(DE3) pLysS [F^−^ *omp*T *hsd*S_B_(r_B_^−^m_B_^−^)*gal dcm* (DE3) pLysS (Cam^R^)] cells by transformation with appropriate plasmid constructs, using standard protocol [9]. pUC19 and M13mp18 phage DNA were isolated as described [9].

### 2.2. Chemicals

Restriction endonucleases and T4 DNA ligase were obtained from New England Biolabs. Phusion DNA polymerase was obtained from Finnzymes. *S*-Adenosyl-L-[^3^H] methionine (AdoMet) (78.9 Ci/mmol) was purchased from PerkinElmer Singapore Pte. Ltd, Singapore, and GE Healthcare Biosciences Ltd., Hong Kong. Calibration kits for molecular weight determination by gel-filtration chromatography analysis were purchased from BioRad Laboratory Inc., USA, and molecular weight markers for SDS-PAGE were purchased from Fermentas Life Sciences, USA. AdoMet, chloramphenicol, ampicillin, bovine serum albumin, HEPES, N-ethylmaleimide, Coomassie brilliant blue, EDTA, RNase A, and S-Adenosyl-L-homocysteine (AdoHcy) were purchased from Sigma-Aldrich Co., USA. Ni-NTA beads were purchased from Invitrogen, USA. Heparin-sepharose was purchased from GE Healthcare Biosciences Ltd. Hong Kong. DE 81 anion-exchange filter papers were purchased from Whatman, USA. ELISA plates were purchased from Thermo Fisher Scientific, Denmark. All other chemicals used were of the highest grade of purity. Oligonucleotides for methylation assays were purchased from the Sigma-Aldrich Co., USA. Double-stranded DNA concentration was measured spectrophotometrically, assuming an A_260_ of 1.0 to correspond to 50 *μ*g/mL for double-strand DNA and 33 *μ*g/mL for single-strand DNA.

### 2.3. Amplification and Cloning of M.EcoP1I

The 1948 bp long *M.EcoP1I* gene was amplified by a polymerase chain reaction (PCR) using pVK1 construct [6] containing a ~2 kb long *EcoP1I mod* gene as a template with Phusion DNA polymerase, using the forward primer and reverse primer (Table 1). The primers were designed with the help of the annotated complete sequence of the P1 prophage by identifying the putative gene sequence of *M.EcoP1I*. The amplified PCR fragment was cloned into the bacterial expression vector pET15b using appropriate restriction enzyme sites to introduce the His_6_-tag at the N-terminus of the M.EcoP1I protein. The DNA construct containing the *M.EcoP1I* gene was confirmed by restriction digestion and sequencing. The construct was named pSN2.

### 2.4. Overexpression and Purification of M.EcoP1I and M.EcoP15I

*E. coli *BL21(DE3) pLysS cells harboring the pSN2 construct were grown at 37°C in an LB broth containing 100 *μ*g/mL ampicillin and 35 *μ*g/mL chloramphenicol to an A_600_ nm ~0.6. M.EcoP1I production was induced by the addition of 0.5 mM IPTG. After three hours of incubation at 37°C, bacterial cells were harvested by centrifugation at 3500 g for 10 minutes. Bacterial cell pellets were resuspended in a sample loading buffer [125 mM Tris–HCl (pH 6.8), 4% SDS, 10% glycerol, 0.06% bromophenol blue, and 25 mM *β*-mercaptoethanol] and lysed by sonication. Inductions were checked by subjecting bacterial cell extracts to 0.1% SDS and 10% PAGE, followed by the staining of protein bands with Coomassie brilliant blue R-250.

Bacterial cells (2 g) were resuspended in 20 mL of buffer A (20 mM Tris-HCl buffer (pH 8.0) containing 500 mM NaCl, 10% glycerol, 2 mM *β*-mercaptoethanol, 2 mM imidazole, 0.5% Triton-X-100, and 1 mM PMSF) and lysed by sonication on ice. The cell lysate was centrifuged at 27,200 g for 1 hour at 4°C, and the supernatant was loaded onto an Ni^2+^-NTA affinity column equilibrated with buffer A. The column was washed with 100 bed volumes of buffer A containing 30 mM imidazole and the protein was eluted with a linear gradient of 100–300 mM imidazole present in the same buffer. Fractions containing the pure (His)_6_-M.EcoP1I protein were pooled and dialyzed against buffer B [10 mM Tris–HCl (pH 7.4) containing 100 mM NaCl, 0.1 mM EDTA, 10% glycerol, and 7 mM *β*-mercaptoethanol] and loaded onto the Heparin-Sepharose column. The column was washed with 10 bed volumes of buffer B and eluted with a linear gradient of 100–500 mM NaCl. Fractions containing the pure protein were dialyzed against a 10 mM Tris pH 7.4 buffer containing 50 mM NaCl and 20% glycerol and stored at 4°C. Protein concentrations were estimated using the Bradford reagent (Sigma-Aldrich Co.) with bovine serum albumin as the standard.

M.EcoP15I was expressed in *E. coli* JM109 by transforming plasmid pUC18-M.EcoP15I [10]. M.EcoP15I was purified as described earlier [10]. The purity of the enzyme was judged as being greater than 99% by SDS-PAGE.

### 2.5. In Vitro Methylation Assays

#### 2.5.1. Filter-Binding Assay

All methylation assays monitored the incorporation of tritiated methyl groups into DNA containing EcoP1I and EcoP15I recognition sequences by using modified ion-exchange filter assays [11]. All data are corrected for the nonspecific binding of [^3^H-methyl]AdoMet to the washed filter. Background counts were measured at zero-time incubation, incubation in the absence of the enzyme was subtracted (less than 100 cpm), and the data were analyzed.

#### 2.5.2. Biotin-Avidin Microplate Assay

A biotin-avidin micro plate assay was used for the analysis of the enzymatic methylation of DNA [12]. The MTase activity of M.EcoP1I was monitored by the incorporation of [^3^H-methyl] groups in biotin-tagged oligonucleotides (duplex I and II) containing its recognition site. The reaction was carried out in 20 *μ*l volume containing a 10 mM Hepes buffer, pH 8.0, containing 6.4 mM MgCl_2_, 0.25 mM EDTA, and 7 mM *β*-mercaptoethanol. Typically, the reactions were performed with 2 pmol of substrate DNA, 100 nM of purified M.EcoP1I, and 1 *μ*M of [^3^H-methyl]AdoMet (78.9 Ci/mmol) at 37°C for 30 minutes.

Microplates were coated with 1 *μ*g avidin dissolved in 100 *μ*l of 100 mM NaHCO_3_ (pH 9.6) and incubated overnight at 4°C. The wells were washed five times with 200 *μ*l PBST (140 mM NaCl, 2.7 mM KCl, 4.3 mM Na_2_HPO_4_, and 1.4 mM K_2_HPO_4_, 0.05% v/v Tween 20, pH 7.2). To measure the activity of the enzyme, the methylation reaction mixture was pipetted into the wells of a micro plate. PBST supplemented with 500 mM NaCl and 1 mM EDTA was added to a total volume of 50 *μ*l and the reaction mixture was incubated for 30 minutes to allow binding of the biotin-tagged oligonucleotides to the micro plate. The wells were washed five times with 200 *μ*l PBST supplemented with 500 mM NaCl to remove the unreacted AdoMet and the enzyme. High salt in the washing buffer was used to prevent binding of the MTase to the DNA. Complete removal of the MTase was important, because unreacted AdoMet could bind to the protein and thereby be retained. Subsequently, the DNA was degraded using 500 ng *Serratia marcescens* endonuclease in 100 *μ*l of 50 mM Tris–HCl, pH 8.0, 5 mM MgCl_2_ for 30 minutes at 37°C. The released radioactivity was estimated by liquid scintillation counting of the reaction mixture after adding 3 mL of scintillation fluid. Each experiment was done in duplicates and repeated three times, and the average values were reported.

### 2.6. Gel-Filtration Analysis of M.EcoP1I

Gel-filtration analysis was performed on a Superose 6 HR 10/30 column in buffer B. To determine the molecular mass of M.EcoP1I, the column was calibrated with suitable molecular weight markers ranging from 12 kDa to 150 kDa. The protein was eluted with the equilibration buffer at 0.2 mL/min and was monitored by measuring the A_280_. The void volume (*V*_o_) of the column was found to be 8 mL and the bed volume 24 mL. The elution volumes (*V*_e_) of marker proteins and M.EcoP1I were determined. The molecular mass of M.EcoP1I was calculated from the plot of *V*_e_/*V*_o_ *versus* log molecular weight.

### 2.7. Isotope Partitioning Analysis

M.EcoP1I (400 nM) was preincubated with 1 *μ*M [^3^H-methyl]AdoMet at 25°C for 5 minutes. The preincubated enzyme was diluted to a final volume of 160 *μ*l, containing labeled 1 *μ*M [^3^H-methyl]AdoMet and 1 *μ*M DNA (duplex I). Aliquots of 20 *μ*l each were removed at 15-, 30-, 45-, 60-, 75-, 90-, 105- and 120-s time intervals and the product formation was analyzed. In a parallel reaction, the abovementioned preincubated mix was brought to 160 *μ*l with a methylation buffer containing 1 *μ*M unlabeled AdoMet and 1.0 *μ*M DNA (duplex I) and the methylation reaction was carried out at 15-, 30-, 45-, 60-, 75-, 90-, 105-, and 120-s-time intervals. Product formation was analyzed using a filter-binding assay.

### 2.8. Determination of Kinetic Parameters

Kinetic studies were done using pUC19 supercoiled plasmid DNA, M13 single-stranded and double-stranded DNA, and oligonucleotide (duplex I) containing the EcoP1I recognition sequence 5′-AGACC-3′. The concentration of DNA was calculated with respect to the number of EcoP1I recognition sites present in it. Methylation assays were carried out for 30 minutes in order to determine initial velocity dependence. In a series of similar reactions containing M.EcoP1I (400 nM) and [^3^H-methyl]AdoMet (1 *μ*M), the concentration of DNA was varied in the range of 50 nM–1 *μ*M. All data are corrected for the nonspecific binding of [^3^H-methyl]AdoMet to the washed filter. Values for *K*_m_^DNA^ and *V*_max_ were found by fitting the data to a rectangular hyperbola in Sigma Plot 9. Similarly, initial velocity experiments were carried out by varying the concentration of [^3^H-methyl]AdoMet in the range of 50 nM–2 *μ*M while keeping the DNA concentration fixed at 1 *μ*M and other reaction conditions identical. Values for *K*_m_^AdoMet^ and *V*_max_ were found by fitting the data to a rectangular hyperbola in Sigma Plot 9. The turnover number (*k*_cat_) was calculated as the ratio of *V*_max_ to the enzyme concentration used. The equations used to obtain the kinetic constants *V*_max_, *K*_m_^DNA^, *K*_m_^AdoMet^, and *k*_cat_ are as described in [13]. Unless otherwise indicated, all enzyme activity data were the average of at least triplicate determinations.

### 2.9. Processivity Studies

A reaction mixture containing a 2 *μ*M biotin-conjugated oligonucleotide containing two EcoP1I sites (duplex II, Table 1) and 250 nM of M.EcoP1I was incubated at room temperature for 5 minutes. After 5 minutes, half of the reaction mixture was removed and 1 *μ*M of [^3^H-methyl]AdoMet was added. Aliquots of 20 *μ*l were withdrawn at different time intervals up to 15 minutes and the reaction was stopped by snap chilling the samples in liquid nitrogen. To the remaining half of the reaction mixture, 1 *μ*M of [^3^H-methyl]AdoMet and 20 *μ*M of a nonbiotin-tagged oligonucleotide (duplex III) containing two EcoP1I sites were added and aliquots of 20 *μ*l were withdrawn at different time intervals. The samples were analyzed using the biotin-avidin micro plate assay. Similar experiments were carried out using M.EcoP15I.

### 2.10. Sulfhydryl Modification of EcoP1I DNA MTase

Aliquots (1 mL) of M.EcoP1I were dialyzed overnight (16 h) at 4°C against a nonreducing buffer (10 mM potassium phosphate (pH 6.8), 0.1 mM EDTA, and 10% glycerol). Following exchange into a nonreducing buffer, the protein concentration was determined using a Bradford protein assay. N-ethylmaleimide (NEM) was dissolved in absolute alcohol and kept at −20°C as a stock solution (200 mM). A given amount of the purified dialyzed M.EcoP1I was incubated at 25°C for 5 minutes with various amounts of NEM in a 10 mM potassium phosphate buffer (pH 6.8) containing 0.1 mM EDTA. After incubation, the reaction mixture was transferred to a methylation assay buffer that contained excess 2-mercaptoethanol to quench the unreacted NEM, and the residual enzyme activity was determined. These experiments were performed three times using different enzyme preparations. The variation in the values was in the range of 3%–5%.

## 3. Results and Discussion

### 3.1. Cloning of EcoP1I DNA Methyltransferase (M.EcoP1I)

*M.EcoP1I* gene was amplified by PCR using primer sequences derived from the 5′- and 3′-ends of the open reading frame and cloned into the pET15b vector. The details of the primers and the restriction sites used for cloning are described in Table 1. *M.EcoP1I* PCR product was cloned into the BamHI site of pET15b to obtain a His-tagged M.EcoP1I. The clones were confirmed by appropriate restriction digestions and the authenticity of the clones was confirmed by DNA sequencing.

M.EcoP1I was heterogeneously overexpressed as an N-terminal His_6_-tagged protein and purified using Ni^++^-NTA chromatography followed by Heparin-Sepharose chromatography. The purified protein was stored in 20% glycerol at 4°C and was found to be active for more than 2 months. Typically, 2 mg of protein of over 95% purity was obtained from 1-liter cultures. M.EcoP1I preparations used for kinetic analysis showed an optimal activity at pH 8.0 and 37°C.

### 3.2. Size-Exclusion Chromatography of M.EcoP1I

**Figure 1:**
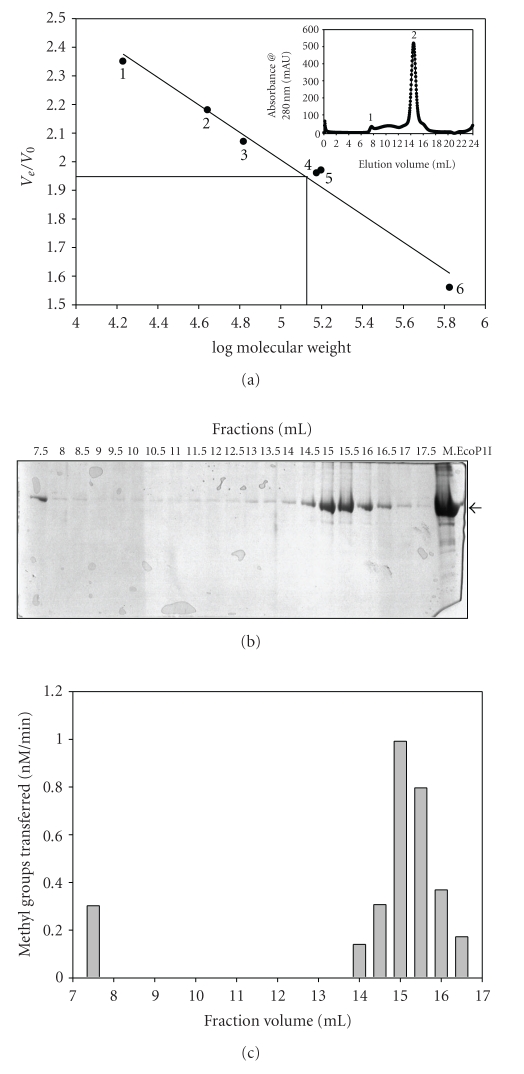
Determination of the molecular mass of M.EcoP1I by size-exclusion chromatography under nondenaturing conditions. (a) The standard curve *V_e_/V_o_* versus log molecular weight was derived from the elution profiles of the standard molecular weight markers with *V_e_* corresponding to the peak elution volume of the protein and *V_o_* representing the void volume of the column determined using Blue dextran (2,000,000 kDa). The peak position of M.EcoP1I is indicated by a line: (1) horse myoglobin (17 kDa); (2) chicken ovalbumin (44 kDa); (3) bovine serum albumin (66 kDa); (4) M.EcoP15I (150 kDa); (5) *γ*-Globulin (158 kDa) and thyroglobulin (670 kDa). Inset: elution profile of M.EcoP1I. (b) Fractions were collected after gel-filtration chromatography and analyzed on SDS-PAGE for presence of M.EcoP1I. (c) Methylation activity of M.EcoP1I present in the fractions was checked using filter-binding assays.

Gel-filtration analysis was performed to determine the size and subunit structure of M.EcoP1I in solution. A superose 6 HR 10/30 column was calibrated with proteins of known size (17–670 kDa), and different concentrations of M.EcoP1I were loaded (3–15 *μ*M). M.EcoP1I was eluted as a symmetric peak at a position corresponding to a globular protein of 150 kDa (Figure 1), suggesting that the enzyme exists mostly as a dimer under native conditions. The elution profile of M.EcoP1I shows an additional small peak which corresponded to a high molecular weight protein (Figure 1(a), inset). The presence of protein in both peaks was confirmed by SDS-PAGE analysis (Figure 1(b)). The protein present in both of the fractions showed MTase activity (Figure 1(c)). Rechromatography of the eluted dimer peak followed a similar pattern; a small peak of high molecular weight protein and a symmetric peak corresponding to the dimer (data not shown). When the peak corresponding to a high molecular weight protein was reloaded onto the column, it was too eluted as two peaks: a high molecular weight protein peak and a dimer peak (data not shown). The elution pattern did not change when M.EcoP1I was loaded in the presence of 500 mM NaCl suggesting a stronger interaction between the monomeric subunits of the protein (data not shown).

Based on the amino acid sequence, the molecular mass of M.EcoP1I was calculated to be ~75 kDa. The molecular mass of purified protein determined by SDS-polyacrylamide gel electrophoresis was ~75 kDa. However, size-exclusion chromatography analysis (Figure 1) clearly showed that M.EcoP1I exists mostly as a dimer in solution in the concentration range of 3–15 *μ*M. It was also eluted as an active high-molecular weight protein peak corresponding to a higher-molecular weight protein. Hornby et al. [14] using equilibrium ultracentrifugation, showed that M.EcoP1I exists as a tetramer (300 kDa) in solution. Later studies using analytical ultracentrifugation showed that M.EcoP1I exists as a dimer in solution [15]. The substrate for most restriction endonucleases (ENases) is unmethylated double stranded DNA while the *in vivo *substrate for a DNA MTase is generally hemimethylated DNA. Hence, it led to the paradigm that most R-M systems comprise dimeric ENases and monomeric MTases. However, an increasing number of studies on DNA MTases show the existence of many dimeric DNA MTases [16]. With a number of published examples of enzyme dimerisation, there do not seem to be any clear cut differences among DNA MTases based on the target residue or mechanism of methyl group transfer. The biological relevance of the dimerisation of DNA MTases needs to be assessed. One possibility is that dimeric MTases may provide stability to enzymes *in vivo* by forming dimers protecting against the premature degradation of the protein. The other possibility is that DNA MTases may bind as dimers to two DNA molecules simultaneously, which allows the formation of a large enzyme-substrate network with high-molecular weight DNA. However, these suggestions are not consistent with dimeric DNA MTases which recognize asymmetric recognition sequences and methylate only one target residue [16]. It is possible that cooperative interactions between a DNA MTase and DNA lead to enzyme dimerisation or oligomerisation on DNA, which is important for the transfer of the methyl group by enzyme to their DNA substrates.

M.EcoP1I exists as a dimer (M_2_) in solution as shown by gel filtration chromatography (Figure 1) whereas EcoP1I restriction enzyme (R.EcoP1I) is known to form a Res_2_-Mod_2_ (R_2_M_2_) complex in solution which is an active endonuclease [15]. The Mod (M_2_) subunit confers recognition sequence specificity. Although, Mod (M_2_) is an independent DNA MTase itself, the Res subunit does not possess any known enzymatic activity. It has been clearly demonstrated that the presence of free Mod (M_2_) subunits along with R_2_M_2_ complex do exist *in vivo* and might be responsible for *in vivo* modification of DNA [4].

### 3.3. Effect of Salt Concentration on M.EcoP1I Activity

**Figure 2:**
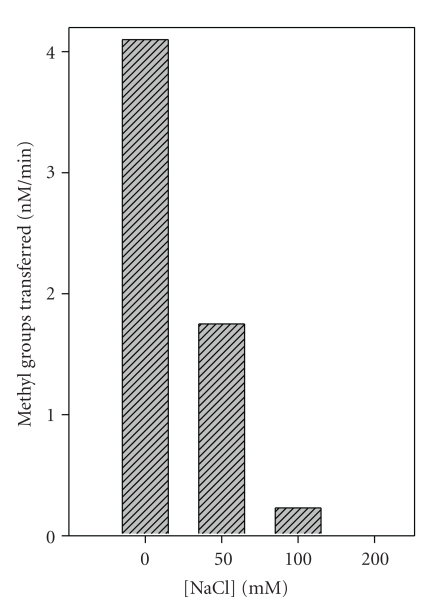
Effect of NaCl concentration on the DNA methylation activity of M.EcoP1I. The effect of NaCl concentration (0–200 mM) on the DNA methylation activity (nM of methyl groups transferred per min) of M.EcoP1I (400 nM) was determined using 1 *μ*M pUC19 plasmid DNA and 1 *μ*M of [^3^H-methyl]AdoMet under standard conditions.

Size exclusion chromatography analysis showed that M.EcoP1I exists mostly as a dimer (Figure 1) and the dimer is stable even at high salt concentrations (data not shown). We therefore checked the methylation activity of M.EcoP1I as a function of ionic strength. As shown in Figure 2, M.EcoP1I methylated DNA only at low salt concentrations. As the salt concentration increased, M.EcoP1I activity was reduced, and in the presence of more than 100 mM NaCl, there was no methylation activity suggesting that though M.EcoP1I is stable at high salt concentrations, it is catalytically inactive. One of the possibilities is that minimum salt concentrations are important for DNA-protein interactions and at high salt concentrations, a weak interaction between DNA and protein results in loss of activity. In the case of *de novo*-type mouse DNA (cytosine-5) MTases (Dnmt3a, Dnmt3b1 and Dnmt3b), a >100 mM NaCl concentration was shown to inhibit the activity [17] whereas PabI DNA MTase showed optimal activity at 200–500 mM NaCl concentrations [18]. EcoDam showed a sharp decrease of methylation activity of unmethylated DNA with an increase in salt concentration. However, the decrease in methylation activity was not very obvious in the case of hemimethylated DNA [19]. The decrease in methylation activity in the presence of high salt concentration indicates that the electrostatic interactions between the enzyme and the DNA phosphate backbone maintain the continued association of the enzyme with the DNA so that methylation occurs.

### 3.4. Preincubation Studies with M.EcoP1I

**Figure 3:**
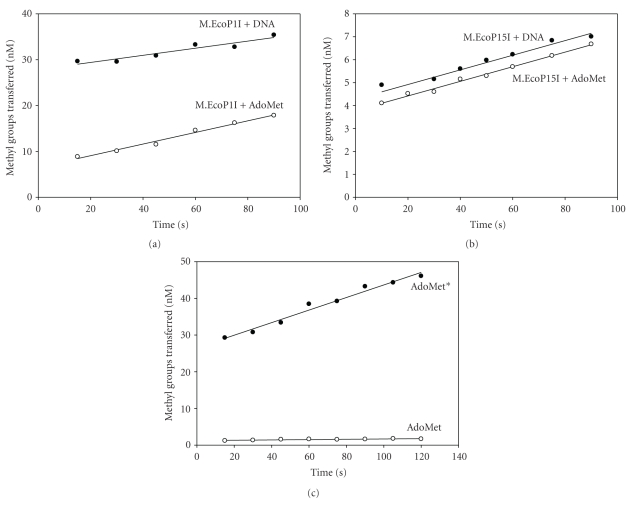
Preincubation analysis of M.EcoP1I and M.EcoP15I. (a) Preincubation analysis of M.EcoP1I. Methylation reactions were carried out in methylation buffer containing 400 nM M.EcoP1I, 1 *μ*M duplex I, or, alternatively, 1 *μ*M [^3^H-methyl]AdoMet. Methylation in the presence of a preformed M.EcoP1I-DNA binary complex (AdoMet added second) is indicated as filled circles (∙). Similarly, a preformed M.EcoP1I-AdoMet binary complex (DNA added second) is shown as empty circles (∘). 20 *μ*l aliquot of the reaction mixture was removed at a 15-second time interval, and the extent of methyl group incorporation was measured. (b) Preincubation analysis of M.EcoP15I (250 nM) with pUC19 DNA (1 *μ*M) and [^3^H-methyl]AdoMet (1 *μ*M). Methylation in the presence of a preformed M.EcoP15I-DNA binary complex (AdoMet added second) is indicated as filled circles (∙). Similarly, a preformed M.EcoP15I-AdoMet binary complex (DNA added second) is shown as empty circles (∘). (c) Isotope partitioning analysis of EcoP1I MTase. Methylation reactions were carried out in methylation buffer containing 400 nM M.EcoP1I, 1 *μ*M duplex I, and 1 *μ*M [^3^H-methyl]AdoMet. Curve 1 (∙, AdoMet*) shows product formation after enzyme was preincubated with 1 *μ*M [^3^H-methyl]AdoMet, and the reaction was started with addition of DNA and labeled [^3^H-methyl]AdoMet. Curve 2 (∘, AdoMet) shows product formation after the enzyme was preincubated with 1 *μ*M [^3^H-methyl]AdoMet and the reaction was started with addition of DNA and unlabeled AdoMet.

For the catalytic cycle of DNA MTases, the binding of substrates could occur in a random or sequential order. To determine this, M.EcoP1I (400 nM) was preincubated with [^3^H-methyl]AdoMet (1 *μ*M) or with duplex I (1 *μ*M) for 5 minutes at 37°C, and the reaction was initiated by adding DNA or [^3^H-methyl]AdoMet, respectively. As shown in Figure 3(a), the preformed enzyme-DNA complex is more efficient than the preformed enzyme-AdoMet complex in methylating DNA. Under saturating substrate conditions, the order of the preincubation of M.EcoP1I with AdoMet and DNA had a significant influence on the rate of product formation.

Similar studies were carried out with EcoP15I DNA MTase, a well-characterized DNA MTase from the Type III R-M system. As can be seen from Figure 3(b), both the preformed enzyme-AdoMet and enzyme-DNA complex gave almost similar rates of methylation indicating that both complexes are catalytically competent to carry out methylation reactions. From these results, it appears that M.EcoP15I binds to substrates in a random bi-bi reaction mechanism. Earlier studies with M.EcoP15I using steady state kinetic studies showed that the methylation reaction proceeds by a random mechanism [20].

From such preincubation experiments, it appears that M.EcoP1I may have a preferred order of substrate binding. The route via the enzyme-DNA complex is kinetically more efficient than that via the enzyme-AdoMet complex. One explanation for this would be that the preformed enzyme-AdoMet complex is catalytically less efficient and that it must dissociate, bind DNA, and then rebind AdoMet, which could slow down a productive complex formation. These results suggest that DNA binds first, followed by AdoMet. Both M.EcoP1I and M.EcoP15I belong to Type III R-M systems and although they share 37% identical amino acids, these DNA MTases carry out methyl transfer reactions possibly using different kinetic mechanisms. While M.EcoRV [21], T4 Dam [22], M.KpnI [23], and M.Eco29kI [24] bound to AdoMet first followed by DNA, EcoDam [25] preferred binding to DNA first followed by AdoMet. In the case of M.BamHI, both the preformed enzyme-DNA and enzyme-AdoMet complex showed almost similar methylation rates under single turnover conditions [26]. The results obtained from preincubation studies need to be further supported by a detailed analysis of the kinetic mechanism under steady state conditions. It is interesting that these MTases, which contain structurally similar domains, have diverse kinetic mechanisms.

### 3.5. Isotope Partitioning Analysis

Isotope partition analysis is used to establish an interaction or binding of the enzyme with the substrate and to study the competency of a binary complex [23]. To confirm the competence of the M.EcoP1I-AdoMet binary complex to methylate DNA, an isotope partitioning analysis was carried out. The enzyme preincubated with labeled [^3^H-methyl]AdoMet was added into reaction mixes containing an excess of DNA and labeled [^3^H-methyl]AdoMet or unlabeled AdoMet. The preincubation of M.EcoP1I with labeled [^3^H-methyl]AdoMet resulted in a burst of product formation upon reaction initiation with the addition of [^3^H-methyl]AdoMet and DNA (Figure 3(c), AdoMet*) followed by a constant rate of product formation. But in the case of chase with unlabeled AdoMet, the incorporation of the [^3^H-methyl] group was much smaller than with labeled [^3^H-methyl]AdoMet (Figure 3(c), AdoMet). If the enzyme-AdoMet complex is catalytically competent, all the bound labeled AdoMet would be transferred to methylate DNA and the rate of product formation would be similar in both cases (with labeled AdoMet and unlabeled AdoMet). This is typical for an ordered bi-bi mechanism when studying the second substrate by isotope partitioning. Because the M.EcoP1I-AdoMet complex is not catalytically competent, the enzyme must dissociate AdoMet and bind with DNA to form a catalytically competent binary complex. These results confirmed that the MTase-AdoMet binary complex is not competent to methylate DNA.

### 3.6. Methylation in Presence of Various Metal Ions

**Figure 4:**
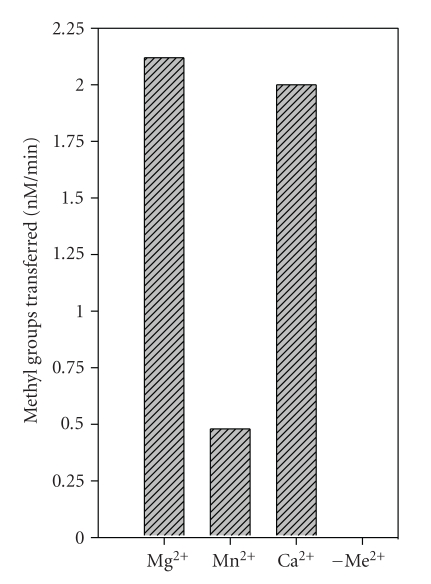
Effect of divalent metal ions on methylation activity of M.EcoP1I. Duplex I (1 *μ*M) and [^3^H-methyl]AdoMet (1 *μ*M) were incubated with M.EcoP1I (400 nM) in the presence of various metal ions (6.4 mM) in methylation buffer. The methylation assay was performed and analyzed using the biotin-avidin micro plate assay as described in Section 2. Metal ions used in the assay are indicated in the histogram. The values represent an average of three determinations and subtracted from background values.

It has been shown that M.EcoP1I methylates DNA only in the presence of Mg^2+^ [4]. To elucidate the effect of various divalent metal ions on methylation activity, methylation assays were performed in the absence and presence of various divalent metal ions. M.EcoP1I did not methylate DNA in the absence of metal ions and methylated DNA in the presence of Mg^2+^, Mn^2+^, and Ca^2+^ (Figure 4). In the presence of Co^2+^, Cu^2+^, Ni^2+^, Cd^2+^, and Zn^2+^, M.EcoP1I did not methylate DNA (data not shown). The enzyme showed maximal activity in the presence of Mg^2+^ and Ca^2+^ whereas in the presence of Mn^2+^, M.EcoP1I methylated DNA less efficiently. It has been shown that M.EcoP1I binds to DNA in the presence of various metal ions with varying affinities [6]. Our results and earlier studies suggest that M.EcoP1 harbors a metal binding center which is important for methylation.

A few DNA MTases require metal ions for their activity whereas the activity of some of the DNA MTases has been shown to be either enhanced or inhibited in the presence of divalent metal ions suggesting that metal ions may play an important regulatory role inside the bacterial cell. Like M.EcoP1I, M.EcoP15I [4], M.AhdI [27], and TspGWI [28] methylated DNA in the presence of metal ions and no methylation activity was seen in the absence of metal ions. The presence of metal ions is not an absolute requirement for M.BcgI [29], M.AloI [30], M.Eco57I [31], and M.MmeI [32] but the methylation activity of these enzymes was enhanced in the presence of Mg^2+^. On the other hand, M.HindIII, M.LlaCI, M.EcoVIII [33], M.PabI [18], M1.MboI, M2.MboII, M1.NcuI, M.NcuII [34, 35], and M.FokI [36] showed inhibition of methylation activity in the presence of various metal ions. It is clear that the activities of DNA MTases characterized so far respond differently in the presence of metal ions.

### 3.7. Dual Metal Ions in Methylation

**Figure 5:**
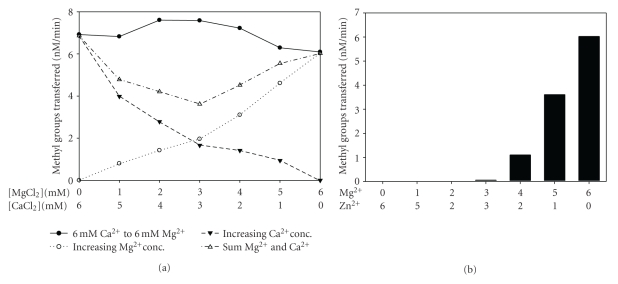
DNA methylation by M.EcoP1I in the presence of two metal ions. (a) Methylation by M.EcoP1I was measured by keeping the total divalent metal ion concentration at 6 mM, that is, from Ca^2+^ (6 mM) on the left to Mg^2+^ (6 mM) on the right (∙). pUC19 supercoiled DNA (1 *μ*M) was incubated with M.EcoP1I (400 nM) in the presence of varying concentrations of metal ions; Ca^2+^ and Mg^2+^ in methylation buffer and assays were performed as described in Materials and Methods. Methylation assays in the presence of Mg^2+^ (∘) and Ca^2+^ (▾) alone are plotted. The plot with open triangle indicated sum of Mg^2+^ and Ca^2+^ concentration dependences. (b) Methylation assays were performed in the presence of Mg^2+^ and Zn^2+^ to a final concentration of 6 mM. pUC19 plasmid DNA (1 *μ*M) was incubated with M.EcoP1I (400 nM) in the presence of varying concentrations of metal ions, Mg^2+^ and Zn^2+^ in methylation buffer without metal ions.

In order to obtain information about the number of metal ions required for enzyme activity, methylation assays were performed in the presence of two metal ions such that the total divalent metal ion concentration was constant with individual metal ion concentrations varying. Since M.EcoP1I has relatively similar levels of activity with Mg^2+^ and Ca^2+^ (Figure 5(a)), activity was measured in the presence of a combination of these two metal ions. Such an approach is useful to gain insights into the number of metal ions involved in the catalysis and has been employed in the case of restriction endonuclease EcoRV [37] and DNA gyrase B [38]. The divalent metal ion concentration (Mg^2+^ and Ca^2+^) was kept constant at 6 mM. The extent of the rate of methylation increased in the presence of Mg^2+^ and Ca^2+^ (Figure 5(a), filled circle with solid line). One explanation for this effect is that the bell-shaped curve represents the sum of the methylation effects of Mg^2+^ and Ca^2+^ concentration-dependence. The methylation in the presence of either Mg^2+^ or Ca^2+^ showed a gradual increase in the rate of the methyl group transferred. The level of enzyme activity when both Mg^2+^ and Ca^2+^ were present was not equal to the sum of the individual metal ion concentration-dependence. This indicates that both metal ions are involved in the methylation reaction in a synergistic manner. To confirm the involvement of possibly more than one metal ion, methylation assays with M.EcoP1I were performed in the presence of Mg^2+^ and Zn^2+^. M.EcoP1I did not show methylation activity in the presence of Zn^2+^ (data not shown). In the presence of both Mg^2+^ and Zn^2+^, inhibition of activity was seen with increasing concentrations of Zn^2+^ (Figure 5(b)). This is in agreement with the earlier observation that Zn^2+^ does not support methylation reactions catalyzed by M.EcoP1I (data not shown). While this result suggests that Zn^2+^ replaces Mg^2+^, it is possible that the affinity for Zn^2+^ is more than that for Mg^2+^ and Ca^2+^. This could explain the requirement of higher concentrations of Mg^2+^ to overcome inhibition in the presence of Zn^2+^. In contrast, a close relative of M.EcoP1I, M.EcoP15I, did not support DNA methylation in the presence of divalent metal ions except Mg^2+^ [8]. A detailed amino acid sequence analysis of M.EcoP1I suggests two possible metal binding sites, the ^358^ID(x)_n_…ExK^399^ and ^600^DxDxD^604^ motifs. Mutation in the putative metal binding motif of M.EcoP1I would reveal the details of a dual metal ion requirement for its activity. It is noteworthy to mention that mutations in the ^357^MD(x)_n_…ExK^377^, Mg^2+^ binding motif of M.EcoP15I resulted in inactive MTases [8].

### 3.8. Initial Velocity Studies with Single-Stranded and Double-Stranded DNA Substrates

Kinetic parameters of methylation were determined using pUC19, M13 mp18 single- and double-stranded DNA and double-stranded oligonucleotides (31 mer) as substrates in the presence of various metal ions such as Mg^2+^, Mn^2+^, and Ca^2+^ under steady state conditions (Table 2). Fitting the data of initial velocity versus substrate concentration to a rectangular hyperbola, the kinetic parameters (*K_m_* and *V*_max_\) were calculated. The data presented in Table 2 indicate that both Mg^2+^ and Ca^2+^ are preferred metal ions for catalysis and that the methylation reaction is slow in the presence of Mn^2+^. M.EcoP1I methylated all DNA substrates with almost similar affinity and comparable turnover numbers in the presence of Mg^2+^ and Ca^2+^ (Table 2). However, in the presence of Mn^2+^, the *K_m_* for all the DNA substrates was at least twofold smaller compared to *K_m_* values in the presence of Mg^2+^ and Ca^2+^. The *k*_cat_ values calculated in the presence of Mn^2+^ were also significantly lower (~10-fold) when compared to *k*_cat_ values in the presence of the other two metal ions. The *K_m_* obtained for AdoMet is 0.5 *μ*M for M.EcoP1I in the presence of Mg^2+^. M.EcoP15I, a well-characterized Type III MTase, showed almost similar *k*_cat_ (0.0053 min^−1^ for 31 bp oligonucleotides substrate and 0.001 min^−1^ for pUC19 plasmid DNA) and *K_m_* (0.8 *μ*M for both 31 bp oligonucleotide and pUC19 plasmid DNA substrates) in the presence of Mg^2+^ [10]. M.EcoP1I is the only MTase from the Type III R-M family so far characterized that has been shown to methylate single-stranded DNA in the presence of various metal ions.

To rule out the self-complementarity of single-stranded DNA, we performed AluI, TaqI, MspI, and XmnI restriction digestions that are very close to EcoP1I recognition site. We did not observe fragmentation of single-strand DNA and DNA was intact after the treatment with restriction enzymes (data not shown). These observations suggest the absence of any self-complementarity of single stranded DNA. EcoP1I DNA MTase (M.EcoP1I) methylates single stranded and double stranded DNA with almost equal affinity. But EcoP1I restriction enzyme (R.EcoP1I) prefers double stranded DNA over single stranded DNA (data not shown).

### 3.9. Processive or Distributive Mode of Methylation

The transfer of methyl groups to DNA by DNA MTases follows either the processive or distributive mode. If the enzyme is processive, it will methylate all the recognition sequences without dissociating from DNA. If the enzyme is distributive, it will dissociate from DNA after one round of catalysis before binding and methylating the second site. To investigate the mode of methylation (processive or distributive) on a DNA substrate containing more than one recognition sequence by M.EcoP1I and M.EcoP15I, a methylation assay was carried out with a biotin-tagged oligonucleotide containing two EcoP1I and EcoP15I recognition sites. The biotin-tagged oligonucleotide was first incubated with a limited concentration of enzyme to form an active enzyme-DNA binary complex. Following preincubation, the reaction was split in two, and to one half, AdoMet was added to start the methylation reaction. To the other half, AdoMet and a 10-fold excess of a nonbiotin-tagged oligonucleotide containing two recognition sites were added to carry out the methylation reaction. The methylation of the biotin-tagged oligonucleotide was then monitored using the biotin-avidin micro plate assay [11]. If M.EcoP1I and M.EcoP15I follow processive mode of methylation, the enzyme will bind to biotin-tagged DNA during preincubation and methylate both the sites before dissociating from it. Therefore, there should be no inhibition of methylation of the biotin-tagged oligonucleotides at different time intervals in the presence of an excess of nonbiotin-tagged oligonucleotides. On the other hand, if these enzymes follow the distributive mode of methylation, after the first round of catalysis on a biotin-tagged oligonucleotide, the enzyme will dissociate from DNA before binding another molecule of DNA for a second round of catalysis. During this dissociation and rebinding, in the presence of an excess of a nonbiotin-tagged oligonucleotide, the enzyme will be trapped and limit the methylation of the biotin-tagged oligonucleotide. Therefore, there will be a difference in methylation of the biotin-tagged oligonucleotide because only one site per DNA molecule will be methylated.

**Figure 6:**
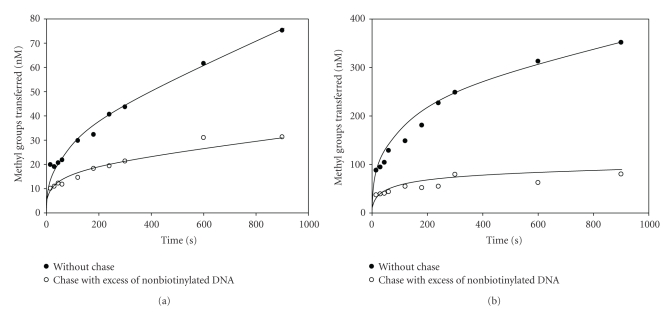
Distributive mode of methylation catalyzed by M.EcoP1I and M.EcoP15I. (a) M.EcoP1I (250 nM) was preincubated at room temperature with 2 *μ*M of biotin-tagged oligonucleotide (duplex II) containing two EcoP1I recognition sites for 5 minutes. After preincubation, the mixture was divided into two sets and to one set; [^3^H-methyl]AdoMet was added to start the reaction (∙). To the other set, [^3^H-methyl]AdoMet was added along with 20 *μ*M of nonbiotin-tagged oligonucleotide (duplex III) to chase the methylation reaction (∘). The reaction was monitored at different time intervals and it indicated and analyzed the extent of methylation of biotin-tagged duplex II using biotin-avidin micro plate assay. (b) M.EcoP15I (250 nM) was preincubated at room temperature with 2 *μ*M of biotin-tagged oligonucleotide (duplex II) containing two EcoP15I recognition sites for 5 minutes. The methylation reaction was performed as described in Materials and Methods and monitored the reaction at different time intervals for methylation of biotin-tagged oligonucleotide (duplex II).

To determine the mode of methylation by M.EcoP1I, a 51 mer oligonucleotide substrate (duplex II) with a biotin-tag at the 5′ end that contained two EcoP1I recognition sites separated by 19 bp was used. The biotin-tagged oligonucleotide (2 *μ*M) was incubated with M.EcoP1I (250 nM) for 5 minutes at room temperature. After the preincubation, the reaction mixture was split in two, to one half, 1 *μ*M of [^3^H-methyl]AdoMet was added to follow the methyl transfer reaction up to 15 minutes. To the other half, 1 *μ*M of [^3^H-methyl]AdoMet and 20 *μ*M of a 51 mer oligonucleotide substrate (duplex III) without a biotin-tag as a trap was followed by methylation up to 15 minutes. The methylation of the biotin-tagged oligonucleotide (duplex II) was followed by a biotin-avidin micro plate assay (Figure 6(a)). In the presence of a 10-fold excess of a nonbiotin-tagged oligonucleotide, the extent of methylation of the biotin-tagged oligonucleotides did not increase, but in the absence of nonbiotin-tagged oligonucleotides, an increase in methylation was observed (Figure 6(a)). To rule out any inhibition of an M.EcoP1I catalyzed methylation reaction in the presence of an excess of DNA, a filter binding assay was carried out with a reaction mixture containing both biotin-tagged and nonbiotin-tagged oligonucleotides. The rate of methylation was almost similar in both conditions suggesting that there was no substrate inhibition in the presence of an excess of DNA (data not shown). These results can be explained only if the DNA is modified by the distributive mode of action. During the distributive mode of the methylation reaction, the enzyme binds to DNA and dissociates from the DNA molecule after one round of catalysis.

To understand the mode of methylation by M.EcoP15I, another well-studied DNA MTase from the Type III R-M system, similar studies were carried out with duplexes II and III containing two 5′-CAGCAG-3′ sequences. As can be seen from Figure 6(b), the rate of methylation of the biotin-tagged oligonucleotide did not increase in the presence of a 10-fold excess of a nonbiotin-tagged oligonucleotide, but in the absence of nonbiotin-tagged oligonucleotides, an increase in the rate of methylation was observed. The results obtained with M.EcoP15I are similar to that of M.EcoP1I, where DNA is modified by the distributive mode of action.

During DNA replication, methylated DNA sequences are converted into a hemimethylated state. The hemimethylated sequences need to be further methylated to maintain the original methylation pattern of DNA. During the methylation reaction, the enzyme binds to DNA nonspecifically and moves along the DNA until it reaches its specific recognition sequence. A processive MTase will stay on the DNA after each methyl group transfer and search for the next target site by linear diffusion. Therefore, many target sites are methylated on the same DNA substrate. In contrast, a distributive MTase would leave the DNA after each methyl group transfer, resulting in many DNA molecules that carry just one or a few methylated sites on the DNA. So far, all the MTases that are accompanied by a restriction endonuclease such as HhaI, HpaII [39], EcoRI [40], and EcoRV [21] show a distributive mechanism of DNA methylation. In contrast, all solitary MTases such as CcrM [41], SssI [39], *E. coli* Dam [25], and Dnmt1 [42] seem to methylate DNA in a processive manner. However, studies with *E. coli* Dam, a solitary DNA MTase, do not support the processive methylation pattern when a DNA segment derived from the regulatory region of the Pap operon was used as a substrate [43]. Recently, Coffin and Reich [19] showed that EcoDam follows intrasite processive catalysis where a single binding event of the enzyme to DNA is sufficient to methylate both the target adenine (one on the top strand and one on the bottom strand) in the GATC sequence. Like many MTases which follow a distributive mechanism of methylation, our study clearly shows that M.EcoP1I methylates DNA containing more than one recognition site in a distributive manner.

We have also carried out experiments with Type III restriction enzymes (R.EcoP1I and R.EcoP15I) to determine the mode of methylation and the results suggest that these Type III restriction enzymes follow a distributive mode of methylation in the absence of ATP (data not shown). In the presence of ATP, Type III restriction enzymes (R_2_M_2_) are more efficient MTases than Mod (M_2_) alone. While ATP stimulates methylation activity of the restriction enzyme, the activity of the M.EcoP1I is not affected. It is proposed that stimulation of methylation activity of R.EcoP1I and R.EcoP15I could be due to DNA translocation which transforms modification from a distributive to a processive reaction.

### 3.10. NEM Modification of EcoP1I DNA MTase

The thiol group of cysteine is one of the most reactive functional groups among all amino acid chains [44]. Of all the available sulfhydryl modifying reagents, N-ethylmaleimide (NEM) has consistently been used for cysteine modification. This is because of its high selectivity for -SH groups. Earlier studies with M.EcoP15I from our group have shown that a cysteine residue in the target recognition domain is important for enzymatic activity. M.EcoP15I contains six cysteine residues at positions 30, 213, 344, 434, 553, and 577 as deduced from the DNA sequence of the *mod *gene. NEM-modified M.EcoP15I was inactive although it bound to DNA efficiently but poorly to AdoMet and it was presumed that cysteine residues present in the close proximity of AdoMet binding motif, FxGxG, (Cys256, Cys456, and Cys461) might have been modified by NEM. However, substitution of Cys344 in M.EcoP15I with alanine resulted in an inactive enzyme which was able to bind AdoMet efficiently but failed to bind DNA. These results suggested that Cys344 is present in DNA binding domain and is important for enzyme activity. Mutational analysis of other cysteine residues did not result in change of enzyme activity [45]. There are eight cysteine residues present in M.EcoP1I at positions at 28, 211, 256, 456, 461, 552, 576, and 615 [46]. Out of these, five cysteine residues are clustered towards the C-terminal region.

**Figure 7:**
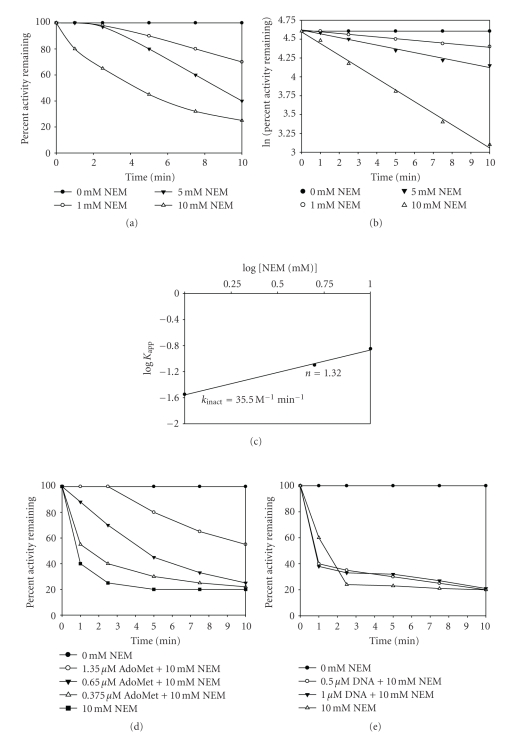
Kinetics of inactivation of M. EcoP1I by N-ethylmaleimide. M. EcoP1I (6.6 *μ*M) was incubated at 25°C in 10 mM sodium phosphate (pH 6.8) containing 0–10 mM NEM. At the indicated times, aliquots were withdrawn and assayed for DNA MTase activity using filter binding assay as described Section 2. (a) Time course of inactivation plot was constructed. Control incubations gave no change in activity. (b) The pseudo-first-order rate constants (*K*_app_) were calculated from the slopes of the inactivation plot for each concentration of NEM used. (c) Pseudo-first-order plot. The apparent first-order rate constants (*K*_app_) were plotted against log[NEM]. The y intercept gives the rate of inactivation of the enzyme (*K*_inact_). The slope of the line gives the number of cysteine residues modified (n). Values are averages of triplicate determinations. (d) M.EcoP1I (6.6 *μ*M) was preincubated with 0.375 *μ*M, 0.65 *μ*M, and 1.35 *μ*M [^3^H-methyl] AdoMet for 10 minutes, followed by addition of 10 mM NEM. Methylation activity of all the samples was analyzed under standard reaction conditions so that the final concentration of AdoMet was the same. (e) M.EcoP1I (6.6 *μ*M) was preincubated with 0.5 *μ*M and 1.0 *μ*M duplex I for 10 minutes, followed by addition of 10 mM NEM. Methylation activities of all samples were analyzed under standard reaction conditions.

To assess the role of cysteines in M.EcoP1I, the enzyme was first dialyzed against a 10 mM potassium phosphate buffer (pH 6.8). This is because NEM is known to modify lysine residues at pH greater than 7, although the reaction is very slow. Inactivation kinetics was carried out with a freshly dialyzed enzyme at 1.0, 5.0, and 10 mM NEM (Figure 7(a)). The modification reaction was arrested by the addition of an excess of 2-mercaptoethanol. The inactivation curves show that only concentrations as high as 10 mM NEM brought about significant inactivation. This suggested that probably a slow reacting cysteine residue was involved in the catalysis. The apparent first-order rate constants (*K*_app_) were calculated from the slopes of the lines obtained from the first-order plot (Figure 7(b)). These values were plotted against the log [NEM] (Figure 7(c)) to obtain a straight line. The slope of this line gave the number of cysteine residues modified. For M.EcoP1I, the value obtained was 1.3, which suggested that a single species of cysteine residues was modified by NEM.

The decrease in enzyme activity could be due to either the modification of a cysteine residue involved in methyl group transfer or because of the modification at the substrate binding sites. To investigate the latter possibility, the enzyme was incubated with AdoMet or the 31 mer duplex I containing the recognition sequence, prior to modification with NEM.  Figure 7(d) shows that partial protection is offered by AdoMet when 10 minutes incubation with AdoMet was followed by addition with 10 mM NEM. When this data was fitted into a second-order plot (data not shown), the protection constant obtained was 1.25 mM. Interestingly, this value is comparable to the AdoMet concentration required to bring about substrate inhibition [20]. The preincubation of the enzyme with an oligonucleotide did not bring about alleviation from inactivation (Figure 7(e)).

Our results with M.EcoP1I indicate that a cysteine residue may be involved in AdoMet binding. The exact cysteine residue that plays a role in catalysis is not known. Sequence alignment of M.EcoP1I with M.EcoP15I showed that cysteine residues at positions 28, 211, 552, and 576 of M.EcoP1I correspond to cysteine residues at positions 30, 213, 553, and 577 of M.EcoP15I. Although Cys256 in M.EcoP1I is not conserved but it is present in target recognition domain as in the case of Cys344 of M.EcoP15I [45]. Taken together, Cys256 may be involved in DNA binding and important M.EcoP1I activity. Further studies involving site-directed mutagenesis need to be done to confirm the role of Cys236 in M.EcoP1I.

The key findings of the present study are the methylation of DNA in the presence of various divalent metal ions and mode of methylation by EcoP1I DNA MTase. Preincubation studies demonstrate an ordered mechanism for M.EcoP1I where DNA binds first followed by AdoMet suggesting that the M.EcoP1I-DNA complex is catalytically competent. M.EcoP1I catalyzes the transfer of methyl groups using a distributive mode of methylation on DNA containing more than one recognition site. The chemical modification of M.EcoP1I using NEM indicates that a cysteine residue may be involved in AdoMet binding. These findings provide an impetus for exploring the role of DNA methylation and metal ions in the structure and function of DNA MTases belonging to Type III R-M systems.

## Figures and Tables

**Table 1:** Primers for amplifying *M.EcoP1I* gene and oligonucleotides as DNA substrates used.

Oligonucleotides	
5′ CGCGGATCCGATGAAAAAAGAAACGATTTTTTCCGAAGTAG 3′	Forward primer
5′ CGCGGATCCTTAGTTCCTTACCACTAAATC 3′	Reverse primer

5′ Bt-TAGGTCAGAATTCAGC**AGACC**CTAAGTAGCC 3′	Duplex I
3′ ATCCAGTCTTAAGTCG**TCTGG**GATTCATCGG 5′	

5′ Bt-CGATGCGACAGC**AGACC**TCTAGTCCAGCGAGACAGC**AGACC**TCTAGAGTCC 3′	Duplex II
3′ GCTACGCTGTCG**TCTGG**AGATCAGGTCGCTCTGTCG**TCTGG**AGATCTCAGG 5′	

5′ CGATGCGACAGC**AGACC**TCTAGTCCAGCGAGACAGC**AGACC**TCTAGAGTCC 3′	Duplex III
3′ GCTACGCTGTCG**TCTGG**AGATCAGGTCGCTCTGTCG**TCTGG**AGATCTCAGG 5′	

AGACC is EcoP1I recognition sequence.

**Table 2:** Kinetic parameters of EcoP1I DNA MTase.

DNA substrate	Mg^2+^		Ca^2+^		Mn^2+^	
	*K*_*m*_ (*μ*M)	*k*_cat_ (min ^−1^)	*K*_*m*_ (*μ*M)	*k*_cat_ (min ^−1^)	*K*_*m*_ (*μ*M)	*k*_cat_ (min ^−1^)
M13 dsDNA	0.42	0.0091	0.25	0.0084	0.15	0.0016
M13 ssDNA	0.31	0.0055	0.29	0.0046	0.14	0.0008
pUC19 DNA	0.31	0.0058	0.45	0.0050	0.21	0.0004
31mer duplex DNA	0.27	0.0066	0.38	0.0058	ND*	ND*

* ND: Not determined.

## Abbreviations

AdoMet: *S*-adenosyl-L-methionine
AdoHcy: *S*-adenosyl-L-homocysteine
ENase: restriction endonuclease
MTase: DNA methyltransferase
R-M: restriction-modification.
